# Stigmasterol alleviates interleukin-1beta-induced chondrocyte injury by down-regulatingsterol regulatory element binding transcription factor 2 to regulateferroptosis

**DOI:** 10.1080/21655979.2021.2000742

**Published:** 2021-11-22

**Authors:** Zhisheng Mo, Peiqing Xu, Huanyu Li

**Affiliations:** Department of Orthopedics, Shenzhen Hospital of Beijing University of Chinese Medicine (Longgang), Shenzhen, P.R. China

**Keywords:** Stigmasterol, knee osteoarthritis, chondrocytes, ferroptosis

## Abstract

Stigmasterol (STM), one of the main active components of *Achyranthes bidentata*, has been shown to effectively inhibit proinflammatory factors and matrix degradation in chondrocytes. However, the effect of STM on interleukin (IL)-1β-induced chondrocytes and its specific mechanism remain unclear. The purpose of the present study was to explore the effect and mechanism of sterol regulatory element binding transcription factor 2 (SREBF2) on IL-1β induced chondrocytes in the presence of STM. CCK-8 was used to detect the effect of STM on the cell viability of mouse chondrogenic cells (ATDC5). After ATDC5 cells were induced by IL-1β, the expression of SREBF2 in osteoarthritis cells was detected by RT-qPCR. The content of iron ion in the cells was detected by using an iron colorimetric assay kit. After further transfection of a SREBF2 overexpressing vector (Oe-SREBF2) or addition of a ferroptosis inhibitor, the expression levels of inflammation and matrix degradation-related proteins were detected via Western blotting. The levels of oxidative stress in cells were determined by using an ELISA kit. The results revealed that STM had no significant effect on the viability of ATDC5 cells. STM reduced IL-1β-induced ATDC5 cell damage and ferroptosis through SREBF2 and enhanced the inhibitory effect of ferroptosis inhibitors on IL-1β-induced ATDC5 cell injury. The present data suggest that STM attenuated chondrocyte injury induced by IL-1β by regulating ferroptosis via down-regulation of SREBF2, and may have potential as a novel therapeutic method for knee osteoarthritis.

## Introduction

Knee osteoarthritis (KOA) is a common and multiple degenerative bone and joint disease, that occurs mainly in the elderly, although epidemiology studies show that the disease gradually tends to affect younger patients [1,2]. With the aging of the global population, the incidence of KOA is increasing year by year [3]. There are numerous pathogenic factors for KOA, but its pathogenesis remains unclear, which hinders patient treatment. Modern medical research shows that KOA is mainly a chronic degenerative disease developed after articular cartilage injury and chondrocyte death, and is often accompanied by cartilaginous ossification and subchondral bone hyperplasia [4,5]. Notably, ferroptosis of chondrocytes is one cause of the gradual decrease of articular chondrocytes during osteoarthritis progression [6].

Previous study has found that *Achyranthes bidentata*, as a Chinese medicine, could effectively treat knee osteoarthritis [7]. Stigmasterol (STM), the active component of *Achyranthes bidentata*, has been found to have a modulating effect on inflammatory factors [8,9]. However, the role of stigmasterol on KOA and its specific mechanism remain unclear. The sterol regulatory element-binding factor-2 (SREBF2) gene is a well-known transcriptional regulator of genes involved in cholesterol biosynthesis. The previous analysis by SwissTargetPrediction database and STITCH database revealed that SREBF2 might be a potential target gene for STM. Meanwhile, SREBF2 gene expression has been reported to be associated with KOA injury, but reports of the specific mechanism remain unclear [10]. Excitingly, SREBF2 could drive the iron homeostasis pathway and thus regulate ferroptosis. Thus, it was inferred that STM might affect ferroptosis by regulating SREBF2 in KOA.

In addition, IL-1β-induced chondrocyte injury has been reported a cellular model for KOA [11,12]. Therefore, the present study aimed to explore the effect and mechanism of STM on IL-1β-induced chondrocytes via SREBF2.

## Materials and methods

### Cell culture and transfection

Mouse chondrogenic cell line (ATDC5 cells) was obtained from the Nanjing Cobioer Biotechnology Co., Ltd., and were cultured with dulbecco’s modified eagle medium (DMEM) (HyClone; Cytiva) supplemented with 10% fetal bovine serum (Gibco; Thermo Fisher Scientific, Inc.) at 37°C in a humid atmosphere with 5% CO_2_. ATDC5 were induced with 10 ng/ml IL-1β or 1 μM ferrostain-1 (Fer-1) to establish an osteoarthritis model *in vitro*. The SREBF2 overexpression lentivirus (Oe-SREBF2) and the negative control (Oe-NC) were purchased from the Shanghai GeneChem Co., Ltd. Transfection experiments were performed using Lipofectamine® 2000 (Invitrogen; Thermo Fisher Scientific, Inc.) according to the operating manual. Cells were harvested 48 h after transfection and transfection efficiency was determined by RT-qPCR.

### Cell counting kit-8 (CCK-8) assays

ATDC5 cells were inoculated in 96-well plates (3 × 10^3^ cells per well) and subsequently incubated with different concentrations of STM (5, 10, 20, 40 μg/ml) for 24 h. 10 μl CCK-8 solution (Solarbio Biotechnology Co., Ltd) was added into each well and incubated for a further 4 h. Next, their absorbance at 450 nm was detected with an enzyme marker (Thermo Fisher Scientific, Inc.).

### Reverse transcription-quantitative PCR (RT-qPCR)

Total RNA was extracted by using the Trizol (Thermo Fisher Scientific, Inc.) according to the manufacturer’s instructions. A reverse transcription kit (Takara Bio, Inc.) was then used to reverse transcribe total RNA into cDNA. Next, an Applied Biosystems™ 7500 Real-Time PCR system (Thermo Fisher Scientific, Inc) was used for cDNA amplification, and 2^−ΔΔCq^ method was employed for analysis of the results [13]. The primers used in were as follows: SREBF2 forward 5’-GCAGCAACGGGACCATTCT-3’ and reverse 5’-CCCCATGACTAAGTCCTTCAACT-3’; GAPDH forward 5’-AGGTCGGTGTGAACGGATTTG-3’ and reverse 5’-GGGGTCGTTGATGGCAACA-3’.

### Western blotting

Total protein was collected with RIPA buffer (Beyotime Institute of Biotechnology), and protein concentration was determined with the BCA protein assay kit (Beyotime Institute of Biotechnology). Proteins were then separated by 10% SDS-PAGE (Beyotime Institute of Biotechnology) and subsequently transferred to PVDF membranes (Millipore, Sigma). Next, the membranes were blocked with 5% skimmed milk and incubated at 4°C overnight with primary antibodies against SREBF2 (1:1000), IL-6 (1:1000), TNF-α (1:1000), IL-10 (1:1000), collagen X (1:1000), MMP13 (1:1000), collagen II (1:1000), aggrecan (1 µg/ml), transferrin receptor protein 1 (TFR1; 1:1000), acetyl coenzyme A synthetase long-chain family member 4 (ACSL4; 1:10,000), glutathione peroxidase 4 (GPX4; 1:1000), solute carrier family 7 member 11 (SLC7A11; 1:1000) and GAPDH (1:500) (all from Abcam). The following day, the membranes were washed with PBS-Tween 20 for three times and then incubated with the secondary antibodies (1:3000) for 2 h. Finally, the immunoreactive signals were detected by Pierce ECL Western blotting Substrate (Thermo Fisher Scientific, Inc.). Semi-quantitative protein expression data was obtained with Image J software (National Institutes of Health).

### Analysis of oxidative stress

Following the manufacturer’s instructions, the corresponding kits (all from Nanjing Jiancheng Bioengineering Institute) were used to analyze the levels of malondialdehyde (MDA), superoxide dismutase (SOD) and glutathione (GSH) released in the supernatant. The samples of each group were quantified with an automatic microplate reader (Syngene).

### Analysis of iron ion levels

The iron ion content in cells was detected using an iron colorimetric assay kit (BioVision, Inc. #K390-100), according to the reagent instructions. Subsequently, absorbance was measured at 593 nm using an enzyme marker.

### Database analysis

Based on the STM structure, the possible binding targets were predicted by the SwissTargetPrediction database (http://www.swisstargetprediction.ch/). Subsequently, the binding of STM to the predicted target was searched and analyzed by STITCH database (Version 5.0, http://stitch.embl.de/).

### Statistical analysis

All the experiments were repeated three times, and the data were analyzed with Graphpad Prism 7.0. (GraphPad Software, Inc.). Data are presented as the mean ± SD. Comparison of multiple groups was evaluated by one-way ANOVA followed by Tukey’s post hoc test. P < 0.05 was considered to indicate a statistically significant difference.

## Result

### Effects of STM on cell vitality

**Figure 1.**
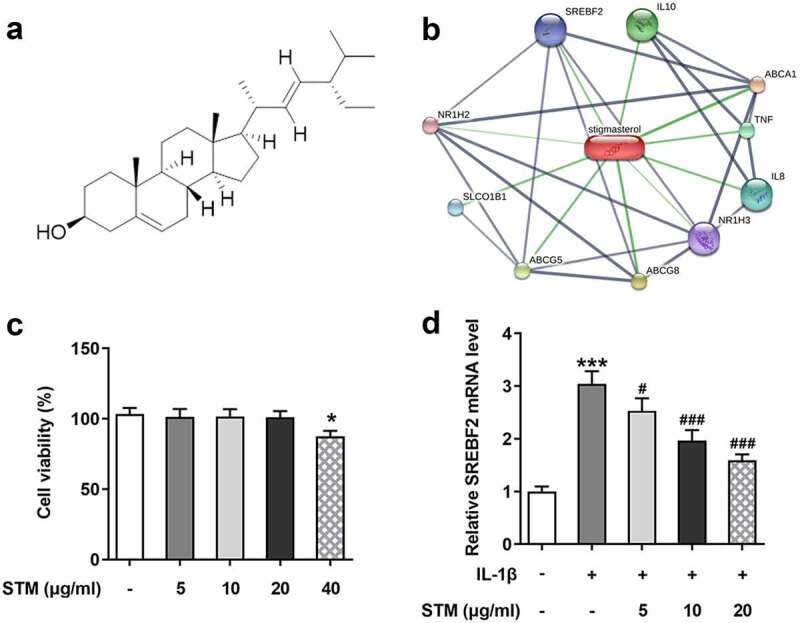
Effects of STM on cell vitality

According to the STM structure (Figure 1(a)), cross-analysis by SwissTargetPrediction and STITCH databases revealed that STM could be combined with SREBF2 (Figure 1(b)). The results of CCK-8 assay showed that 0–20 μg/ml STM had no significant effect on the vitality of ATDC5 cells (Figure 1(c)). But 40 μg/ml STM showed an inhibitory effect on cell viability. Hence subsequent studies were conducted using 0–20 μg/ml STM. To further explore the effect of STM on chondrocyte damage caused by IL-1β, 10 ng/ml IL-1β was used in the present study to induce mouse chondrocyte ATDC5 cells and establish an *in vitro* osteoarthritis model. The RT-qPCR results showed that, after the induction of IL-1β in ATDC5 cells, the expression of SREBF2 was up-regulated. Upon addition of STM, the SREBF2 mRNA expression was down-regulated with an increase in the concentration of STM (Figure 1(d)). Since 20 μg/ml STM showed the greatest difference, this concentration was chosen for subsequent experiments to facilitate the study.

(a) Chemical structure of STM. (b) The target genes of STM were analyzed using the STITCH database. (c) Cell Counting Kit-8 assay was used to determine the viability of ATDC5 cells. (d) The SREBF2 mRNA expression was detected by RT-qPCR. *p < 0.05 and *** p < 0.001 vs. Control; ^#^p < 0.05 and ^###^p < 0.001 vs. IL-1β.

### STM reduces cell injury in IL-1β -induced ATDC5 cells via SREBF2

**Figure 2.**
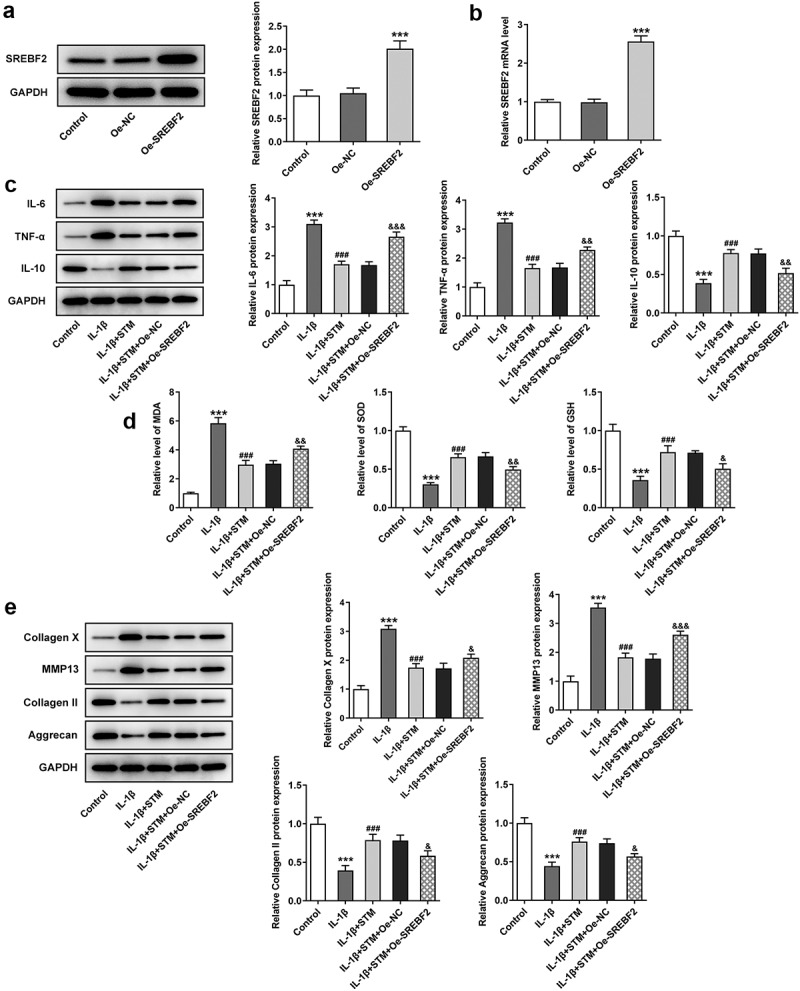
STM reduces cell damage in IL-1β-induced ATDC5 cells via SREBF2

The Oe-SREBF2 ATDC5 cells were constructed by transfection, and the results of RT-qPCR and Western blotting revealed that the mRNA and protein expression levels of SREBF2 were significantly increased in Oe-SREBF2 ATDC5 cells (Figure 2(a-b)). Compared with those of the IL-1β group, the protein levels of IL-6 and TNF-α were decreased, while the IL-10 level was markedly elevated in the IL-1β+STM group. But, overexpression of SREBF2 could reversed this trend (Figure 2(c)). Conversely, upregulation of MDA, as well as downregulation of SOD and GSH, was observed in IL-1β group compared with the findings in the control group (Figure 2(d)). Compared with IL-1β group, the MDA levels was attenuated and the SOD and GSH levels were increased in the IL-1β+STM group. However, the trends of MDA, SOD and GSH were reversed again in the IL-1β+STM+Oe-SREBF2 group compared to the IL-1β+STM+Oe-NC group (Figure 2(d)). Furthermore, Western blotting was used to detect the expression of proteins associated with extracellular matrix degradation (Figure 2(e)). After induction of ATDC5 cells by IL-1β, the expression levels of collagen X and MMP13 were significantly increased, while those of collagen II and aggrecan were significantly decreased. Following STM treatment, the expression levels of collagen X and MMP13 were significantly down-regulated, while those of collagen II and aggrecan were significantly increased. Overexpression of SREBF2 could reverse this trend (Figure 2(e)).

(a) The SREBF2 protein expression was detected by Western blotting. (b) The SREBF2 mRNA expression was detected by RT-qPCR. (c) The protein levels of IL-6, TNF-α and IL-10 were detected by using Western blotting. (d) The levels of MDA, SOD and GSH were detected by using the corresponding kits. (e) The expression levels of collagen X, MMP13, collagen II and aggrecan were detected by Western blotting. ***p < 0.001 vs. Control; ^###^p < 0.001 vs. IL-1β; ^&^p < 0.05, ^&&^p < 0.01 and ^&&&^p < 0.001 vs. IL-1β+STM+Oe-NC.

### STM reduces ferroptosis in IL-1β-induced ATDC5 cells via SREBF2

**Figure 3.**
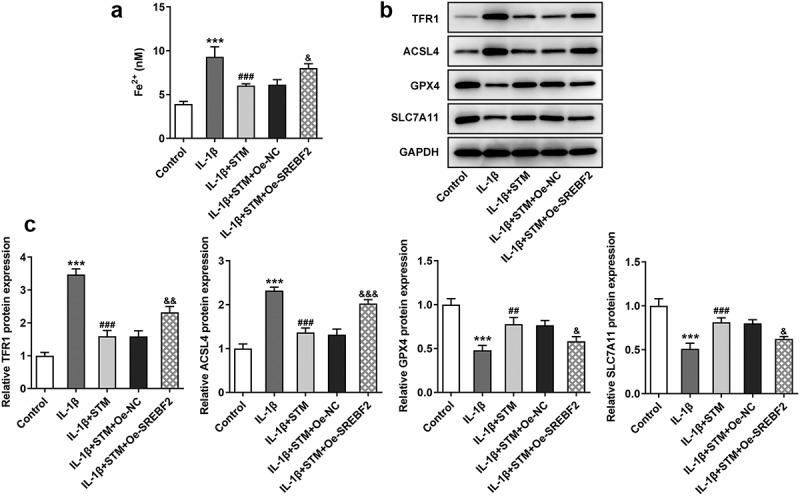
STM reduces ferroptosis in IL-1β-induced ATDC5 cells via SREBF2

The iron content in cells was detected using an iron colorimetric assay kit (Figure 3(a)). The concentration of Fe^2+^ increased after IL-1β induction (IL-1β vs. control). Fe^2+^ concentration decreased after STM treatment (IL-1β+STM vs. IL-1β), while it increased in the IL-1β+STM+Oe-SREBF2 group (IL-1β+STM+Oe-SREBF2 vs. IL-1β+STM+Oe-NC). Western blotting was used to detect the expression levels of ferroptosis-related proteins. The expression levels of TFR1 and ACSL4 were significantly decreased, while those of GPX4 and SLC7A11 were significantly increased in the IL-1β+STM group, compared with the findings in the IL-1β group. The overexpression of SREBF2 could reversed this trend (Figure 3(b-c)).

(a) The iron content in cells was detected using an iron colorimetric assay kit. (b) The expression levels of TFR1, ACSL4, GPX4 and SLC7A11 were detected by using Western blotting. (c) The semi-quantitative results of the expression levels of TFR1, ACSL4, GPX4 and SLC7A11. ***p < 0.001 vs. Control; ^##^p < 0.01 and ^###^p < 0.001 vs. IL-1β; ^&^p < 0.05, ^&&^p < 0.01 and ^&&&^p < 0.001 vs. IL-1β+STM+Oe-NC.

### STM enhances the inhibition of the ferroptosis inhibitor in IL-1β-induced ATDC5 cells

**Figure 4.**
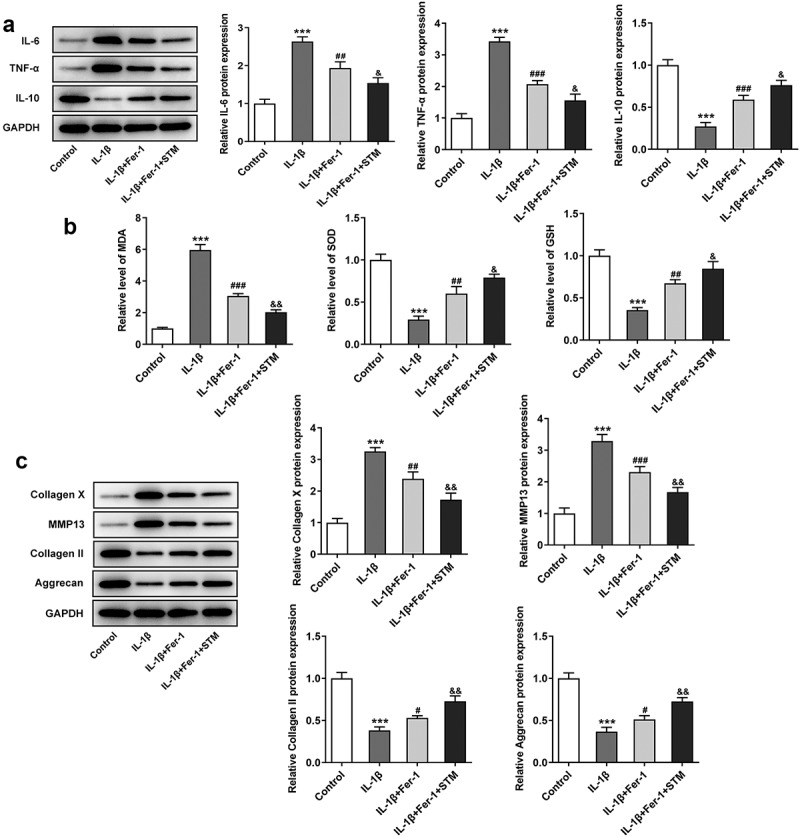
STM enhances the inhibition of the ferroptosis inhibitor in IL-1β-induced ATDC5 cells

To further explore the effect of TSM on IL-1β-induced ATDC5 cell damage, chondrocytes were treated with a ferroptosis inhibitor. Briefly, 1 μM Fer-1 was added to the cell medium. Fer-1 significantly reduced the expression levels of the inflammation-related proteins IL-6 and TNF-α, which were further reduced after the addition of STM (Figure 4(a)). In addition, compared with those in the IL-1β group, the expression levels of SOD and GSH were up-regulated in the IL-1β+Fer-1 group, and were further up-regulated after the addition of STM. The expression of MDA exhibited the opposite trend (Figure 4(b)). Western blot analysis of extracellular matrix degradation-related proteins showed that, compared with those in the IL-1β group, the protein levels of collagen X and MMP13 decreased, while the protein levels of collagen II and aggrecan increased (IL-1β+Fer-1 vs. IL-1β). After the addition of STM, the protein expression levels of collagen X and MMP13 were further reduced, while those of collagen II and aggrecan were further increased (IL-1β+Fer-1+ STM vs. IL-1β+Fer-1) (Figure 4(c)).

(a) The protein levels of IL-6, TNF-α and IL-10 were detected by using Western blotting. (b) The levels of MDA, SOD and GSH were detected by using the corresponding kits. (c) The expression levels of collagen X, MMP13, collagen II and aggrecan were detected by Western blotting. ***p < 0.001 vs. Control; ^#^p < 0.05, ^##^p < 0.01 and ^###^p < 0.001 vs. IL-1β; ^&^p < 0.05 and ^&&^p < 0.01 vs. IL-1β+Fer-1.

## Discussion

To investigate the effect and mechanism of STM on IL-1β-induced chondrocytes, the potential target genes of STM were analyzed using the SwissTargetPrediction database and the STITCH database, and it was found that the common genes in the two datasets were SREBF2 (SREBP2), nuclear receptor subfamily 1 group H member 3 (NR1H3) and NR1H2 [14]. Notably, the previous study has found that STM could inhibit the expression of SREBF2 [15]. The expression of SREBF2 was upregulated in osteoarthritis and played an important role in the pathogenesis of this disease [6,15]. SREBF2 could also directly induce the transcription of transferrin [16]. In addition, there has been a proven association of ferroptosis with cerebral hemorrhage, traumatic brain injury, carcinogenesis and many degenerative diseases (e.g., Alzheimer’s disease, Parkinson’s disease) [17–20]. Of interest, based on the fact that osteoarthritis has features common to ferroptosis such as abnormal iron metabolism, lipid peroxidation and mitochondrial dysfunction, Yao et al. confirmed the possible involvement of ferroptosis in the progression of osteoarthritis and suggested that ferroptosis could be one of the strategies to regulate osteoarthritis [6]. Therefore, it is considered that STM might alleviate KOA by regulating ferroptosis via downregulation of SREBF2.

Research has shown that chondrocytes are the only cellular component of cartilage tissue involved in synthesizing extracellular matrix components and providing matrix turnover, which was essential to maintain the functional and structural integrity of cartilage [21,22]. However, in osteoarthritis, the dynamic balance between cartilage matrix synthesis and degradation was destroyed by stress-induced inflammatory mediators [21,23]. Among the inflammatory factors, IL-1β adversely affected chondrocytes via impairing cell viability and inhibiting synabolic processes associated with cartilage homeostasis [24,25]. Thus, IL-1β induction in chondrocytes has been used as a routine method for generating osteoarthritis models *in vitro* [24–26]. In the present study, in IL-1β-induced ATDC5 cells, the expression of inflammatory factors IL-6 and TNF-α was enhanced; oxidative stress-related MDA levels were increased and SOD and GSH levels were decreased; in the other, the protein expressions of collagen X and MMP13, which are associated with extracellular matrix degradation, were upregulated, and the those of collagen II and aggrecan were downregulated. More importantly, iron ions were increased after IL-1β induction and there were remarkable alterations in the expression of ferroptosis-related proteins. These results again suggest that the IL-1β-induced chondrocyte model was consistent with the phenotype produced by the cells in KOA [27]. So, the present study investigated the effect of STM on IL-1β-induced cell injury and ferroptosis in ATDC5 cells via SREBF2 to confirm the above hypothesis.

The research found that STM reduces the release of inflammatory factors from ATDC5 cells induced by IL-1β through SREBF2, and reduces the level of oxidative stress induced by IL-1β in ATDC5 cells, which indicates the protective effect of STM on cells. This corresponds to previous reports that STM could block cartilage degradation and protect against collagen-induced arthritis [28]. And it has been documented that in IL-1β-treated chondrocytes, STM could effectively inhibit the degradation of pro-inflammatory factors and matrix, as well as suppress the IL-1β-induced NF-κB signaling pathway [29]. In addition, Fotini et al. demonstrated the association between SREBP-2 and the pathogenesis of osteoarthritis, and provided evidence of the relevant molecular mechanisms [30]. The authors suggested that TGF-β induces the activation of the SREBP-2 pathway through integrin subunit α V and PI3K, thus playing a key role in osteoarthritis, and that integrin blockage may be a potential molecular target for osteoarthritis therapy [30]. This study showed that the expression of SREBF2 was upregulated in IL-1β-inducted chondrocyte ATDC5 cells, while its expression was suppressed after STM treatment. Overexpression of SREBF2 could reverse the effect of STM on IL-1β-induced ATDC5 cells, which further indicates that STM reduces IL-1β-induced chondrocyte ATDC5 cell damage through SREBF2. Excitingly, it was found that the inflammatory response and oxidative stress were markedly inhibited in IL-1β-induced chondrocytes while the regulation of extracellular matrix degradation-associated proteins was evident upon the combined action of STM and ferroptosis inhibitors. This suggests that STM might enhance the effect of ferroptosis inhibitors on IL-1β-induced chondrocyte injury, further providing a novel idea for KOA treatment.

## Conclusion

Taken together, the present study has demonstrated that STM attenuates chondrocyte injury induced by IL-1β by regulating ferroptosis via down-regulation of SREBF2, which suggests that STM may have potential as a novel therapeutic method for KOA.

## Highlights

(1). Stigmasterol reduces cell damage in IL-1β-induced chondrocyte injury via SREBF2.

(2). Stigmasterol reduces ferroptosis in IL-1β-induced chondrocyte injury via SREBF2.

(3). Stigmasterol enhances the inhibition of the ferroptosis inhibitor in IL-1β-induced chondrocyte injury.
